# Burden of Healthcare-Associated Infections in Sicily, Italy: Estimates from the Regional Point Prevalence Surveys 2016–2018

**DOI:** 10.3390/antibiotics10111360

**Published:** 2021-11-08

**Authors:** Martina Barchitta, Andrea Maugeri, Maria Clara La Rosa, Claudia La Mastra, Giuseppe Murolo, Giovanni Corrao, Antonella Agodi

**Affiliations:** 1 Department of Medical and Surgical Sciences and Advanced Technologies “GF Ingrassia”, University of Catania, 95123 Catania, Italy; martina.barchitta@unict.it (M.B.); andrea.maugeri@unict.it (A.M.); mariclalarosa@gmail.com (M.C.L.R.); claudia.lamastra@outlook.it (C.L.M.); 2 Regional Health Authority of the Sicilian Region, 90133 Palermo, Italy; giumurolo@gmail.com; 3 National Centre for Healthcare Research and Pharmacoepidemiology, University of Milano-Bicocca, 20126 Milan, Italy; giovanni.corrao@unimib.it; 4 Unit of Biostatistics, Epidemiology and Public Health, Department of Statistics and Quantitative Methods, University of Milano-Bicocca, 20126 Milan, Italy; 5 Azienda Ospedaliero Universitaria Policlinico “G. Rodolico-San Marco”, 95123 Catania, Italy

**Keywords:** DALYs, hospital-acquired infections, prevalence study, pneumoniae, bloodstream infection, surgical site infection, urinary tract infection, BCODE

## Abstract

An assessment of the burden of healthcare-associated infections (HAIs) in terms of disability-adjusted life years (DALYs) is useful for comparing and ranking HAIs and to support infection prevention and control strategies. We estimated the burden of healthcare-associated pneumoniae (HAP), bloodstream infection (HA BSI), urinary tract infection (HA UTI), and surgical site infection (SSI) in Sicily, Italy. We used data from 15,642 patients aged 45 years and above, identified during three repeated point prevalence surveys (PPSs) conducted from 2016 to 2018 according to the European Centre for Disease Prevention and Control protocol. The methodology of the Burden of Communicable Diseases in Europe project was employed. The selected HAIs accounted for 8424 DALYs (95% uncertainty interval (UI): 7394–9605) annually in Sicily, corresponding to 344 DALYs per 100,000 inhabitants aged 45 years and above (95% UI: 302–392). Notably, more than 60% of the burden was attributable to HAP, followed by HA BSI, SSI, and HA UTI. The latter had the lowest burden despite a relatively high incidence, whereas HA BSI generated a high burden even through a relatively low incidence. Differences between our estimates and those of European and Italian PPSs encourage the estimation of the burden of HAIs region by region.

## 1. Introduction

Amongst all the issues that public health must face, healthcare-associated infections (HAIs) and antimicrobial resistance (AMR) have a great impact in terms of incidence, attributable mortality, increased hospital stays, and assistance costs in the hospital setting [1,2,3]. The surveillance—defined as the systematic collection, analysis, and interpretation of health data—is considered by the World Health Organization (WHO) as one of the core components for effective infection prevention and control (IPC) programs against HAIs and AMR [3]. In fact, surveillance is important to identify problems and priorities, providing a benchmark for analyzing trends within and between hospitals [4]. It is also for this reason that the European Centre for Disease Prevention and Control (ECDC) promoted and coordinated the first European point prevalence survey (PPS) of HAIs and antimicrobial use in acute care hospitals in 2011–12, which was then repeated in 2016–17 [4,5]. According to the European 2016–17 PPS, ~6% of all patients staying in acute care hospitals on a given day were infected with at least one HAI [4,5]. As a part of the European PPS, it was also estimated that nearly 8% of patients in Italian acute care hospitals had at least one HAI on the day of the survey [6]. At a regional level, instead, a recent analysis of the three repeated PPSs conducted in Sicily, Italy from 2016 to 2018 estimated approximately 5% prevalence of HAIs, without a significant trend from year to year [7]. By contrast, the same study showed an increasing trend in the use of antibiotics from 2016 to 2018 [7].

For a better estimation of the burden of HAIs, the Burden of Communicable Diseases in Europe (BCoDE) project developed a methodology that considers the years lived with disabilities (YLDs) following the onset of an infection and the years of life lost (YLLs) due to premature mortality. The sum of these measures results in a composite health indicator, the so-called disability-adjusted life year (DALY) [8]. Based on this methodology, the burden of healthcare-associated pneumonia (HAP), urinary tract infection (HA UTI), surgical site infection (SSI), Clostridium difficile infection, neonatal sepsis, and primary bloodstream infection (HA BSI) in Europe were investigated by Cassini et al. [9]. The corresponding estimates of 2.5 million DALYs were, for the most part, caused by HAP and HA BSI [9]. After that, the authors estimated the burden of five types of infections with 16 antibiotic-resistant bacterium combinations. Interestingly, these infections accounted for approximately 30,000 attributable deaths and 900,000 DALYs, with the highest burden in Italy and Greece [10]. More recently, Bordino et al. employed the same methodology to estimate the burden of HAIs in Italy from the 2016 national PPS data. Notably, the total annual DALYs were nearly 425,000 corresponding to 703 DALYs per 100,000 inhabitants [11].

Yet, in a country like Italy where the decentralized healthcare system and regional administrations play a crucial role in IPC programs, it is important to assess the burden of HAIs region by region. Sicily is one of the five autonomous regions in Italy and has almost 5 million inhabitants, of which approximately 51% are women and 50% over 45 years. According to the latest available data in 2019, there are 67 public hospitals and 59 accredited healthcare facilities in Sicily, with a total of 3.1 beds per 1000 inhabitants [12]. The first regional PPS of HAIs and antimicrobial use in acute care hospitals was launched in 2016, which was followed by two further editions in 2017 and 2018 [13], in the framework of a regional action plan for the prevention of HAIs, AMR, and inappropriate use of antimicrobials. The Sicilian PPSs adopted the same protocol and definitions used for the European and Italian PPSs [14]. In the present study, we applied the BCoDE methodology to estimate the burden of the four most frequent HAI types (i.e., HAP, HA BSI, HA UTI, and SSI) in Sicily, using data from the three Sicilian PPSs. We restricted our analysis to patients aged 45 years or older because they accounted for 90% of all enrolled patients. The small number of patients younger than 45 years and hence the low or null number of events in some age- and sex-specific strata would have produced inaccurate estimates of the burden of HAIs.

## 2. Results

**Figure 1:**
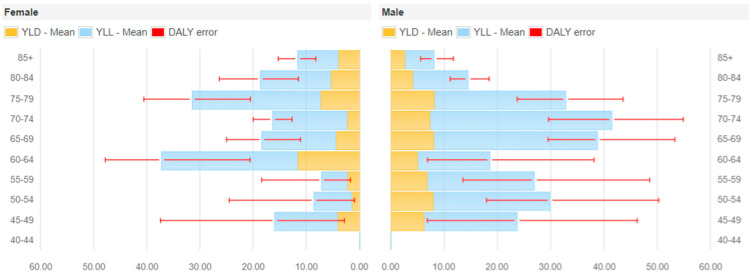
Estimated annual burden of selected HAIs on DALYs per 100,000 inhabitants aged 45 years and older (median and 95% uncertainty interval) by gender and age group, split between YLLs and YLDs.

Based on Sicilian PPSs data on 15,642 patients enrolled from 2016 to 2018, we estimated that a total of 22,676 (95% UI: 21,444 to 23,900) new cases of the four types of HAIs under study occur every year in Sicily among people aged 45 years and above. These HAIs accounted for a total of 8424 DALYs (95% UI: 7394 to 9605) annually, consisting of 6331 YLLs (5529 to 7273) and 2092 YLDs (95% UI: 1811 to 2401). These estimates corresponded to 344 DALYs per 100,000 inhabitants aged 45 years and above (95% UI: 302 to 392). Figure 1 shows the distribution of DALYs per 100,000 inhabitants aged 45 years and above by gender and age categories. In particular, 58.4% of DALYs were attributable to men, with the highest burden among 70–74 year old men and 60–64 year old women. When applying a 3.5% annual time discount rate, the HAIs accounted for 6296 DALYs (5599 to 7064), corresponding to 257 DALYs per 100,000 inhabitants aged 45 years and above (95% UI: 229 to 289).

**Figure 2:**
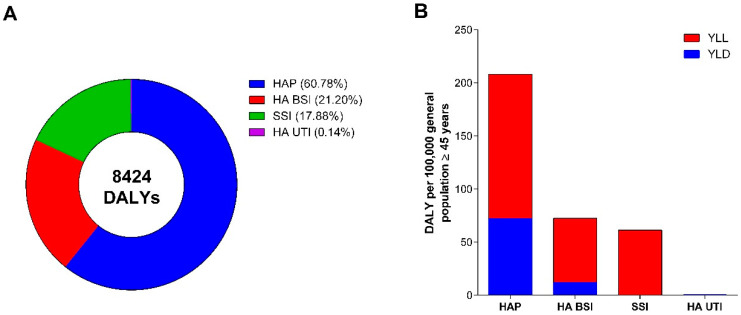
Estimated annual burden in DALYs by different types of HAI: (**A**) The proportion of the total burden on DALYs for each of the selected HAI types; (**B**) Estimated annual burden in DALYs per 100,000 inhabitants aged 45 years and older by different HAI types, split between YLLs and YLDs.

As depicted in Figure 2A, more than 60% of the total burden of the four selected types of HAIs was accounted for by HAP, followed by HA BSI, SSI, and HA UTI. Notably, Figure 2B shows that HAP accounted for a total of 208 DALYs per 100,000 inhabitants aged 45 years and older (95% UI: 176 to 244), which consisted of 136 YLLs (114 to 160) and 72 YLDs (60 to 85). Both HA BSI and SSI contributed to a lesser extent to the total burden of HAIs, respectively with 73 (95% UI: 45 to 112) and 61 (95% UI: 53 to 70) DALYs per 100,000 inhabitants aged 45 years and older. By contrast, HA UTI accounted for a relatively low burden, corresponding to less than 1% of the total DALYs.

In line with these estimates, 677 deaths (95% UI: 619 to 738) each year in Sicily were attributable to the selected HAI types among people aged 45 years and older. Notably, 56% of the estimated deaths were attributable to HAP, 25% to HA BSI, and 19% to SSI.

The detailed results for each type of HAI (aggregate and stratified by the McCabe score) are reported in the Supplementary File S1. For each of the four types of HAI under study, the McCabe 1 group was the one with the highest burden in terms of DALYs per 100,000 inhabitants aged 45 years and older: 182.7 DALYs for HAP, 67.5 for HA BSI, 59.6 for SSI, and 0.2 for HA UTI. This was probably due to the higher incidence of HAIs in these patients and to their longer life expectancy. In terms of deaths, the highest burden was attributable to HAP among patients with McCabe 1 (7.0 attributable deaths per 100,000 inhabitants aged 45 years or over; 95% UI: 4.4 to 10.5).

**Figure 3:**
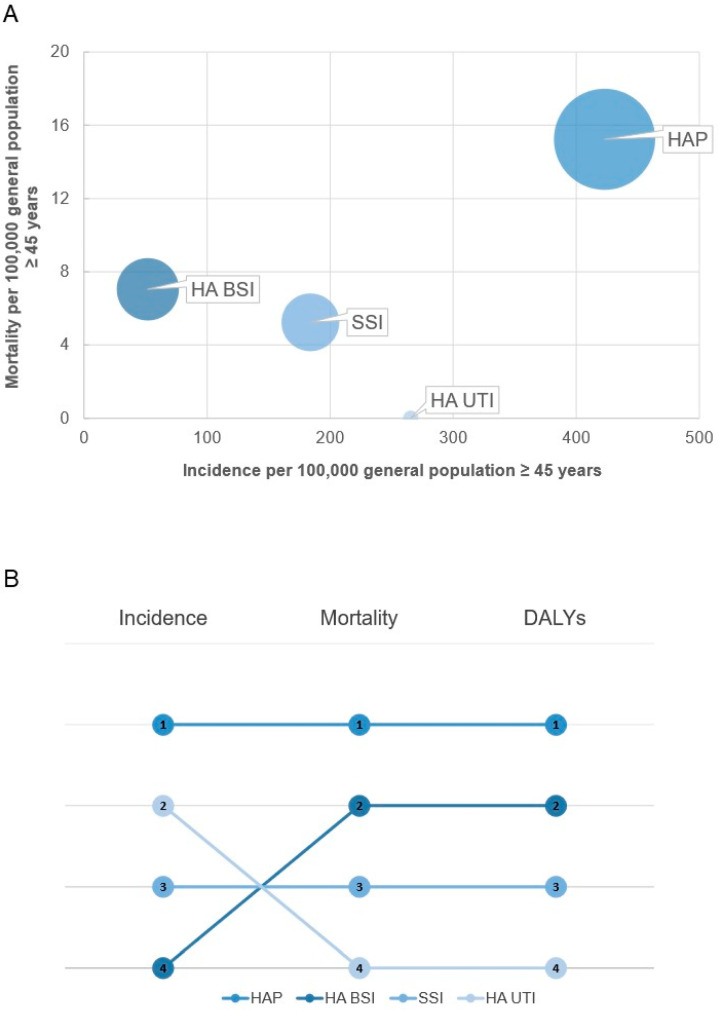
The relationship between incidence, mortality, and DALYs for each of the selected types of HAI: (**A**) Bubble chart representing the incidence per 100,000 inhabitants aged 45 years and older (*x*-axis), the mortality per 100,000 inhabitants aged 45 years and older (*y*-axis), and DALYs per 100,000 inhabitants aged 45 years and older (width of bubble); (**B**) Ranking of the selected HAIs according to their incidence, mortality, and DALYs.

The bubble chart in Figure 3A illustrates the relationship between incidence, mortality, and DALYs per 100,000 inhabitants aged 45 years and above for each type of HAI. More specifically, Figure 3B summarizes how the ranking of selected HAIs varied depending on which indicator was used. As shown, HAP generated the highest burden due to their high incidence and mortality, whereas HA UTI had the lowest burden despite a relatively high incidence. By contrast, HA BSI generated a high burden due to their high attributable mortality even with a relatively low incidence.

## 3. Discussion

Using data from three repeated regional PPSs, we estimated that the burden of HAIs in Sicily (Italy) was 344 DALYs (95% UI: 302 to 392) per 100,000 people aged 45 years or older. In our study, the estimate of the burden of HAIs was lower than that obtained from the Italian (703 DALYs per 100,000 general population, 95% UI: 575 to 845) and the European PPSs (501 DALYs per 100,000 general population, 95% UI: 429 to 582) [9,11]. Furthermore, the comparison with national and international results based on the impact of each type of HAIs on the total burden provides an interesting insight. Particularly, Bordino et al. showed that HA BSI had the highest burden (59% of the total DALYs), followed by HAP (29%). Cassini et al. estimated that more than 60% of the total DALYs was accounted for by HAP and HA BSI (33.7% and 28.9%, respectively) [9,11]. In our study, HAP had the highest burden among the four selected HAIs, accounting for nearly 60% of the total DALYs, while 21% of the burden was attributable to HA BSI [9]. Comparison of results of the burden of HAIs by gender showed that in our study approximately 58% of the total DALYs was attributable to men, a proportion that was similar to that observed by Cassini et al. (59%) and by Bordino et al. (56%) [9,11]. Our results report that the age-strata with the highest burden were 60–64 years for women and 70–74 years for men. In the European PPS the age-strata with the highest burden were 70–74 years for women and 55–59 years for men [9] and in the Italian PPS, instead, the age-stratum with the highest burden was 70–74 years for both men and women [11]. However, it is important to highlight a peculiarity of our regional-specific surveys. In the European and in the Italian PPSs, representative samples of hospitals were selected using a systematic sampling design. Conversely, in the three Sicilian PPSs all acute care hospitals were invited to participate in the framework of a regional action plan for the prevention of HAIs, AMR, and inappropriate use of antimicrobials [7]. We also recognize that some of these differences were probably due to the fact that in our study we limited the analysis to four selected HAIs (i.e., HAP, HA BSI, SSI, and HA UTI) and to patients aged 45 years or older. However, it should also be underlined that the prevalence of HAIs in Sicily was slightly lower than in Europe [4], Italy [6] and in one other Italian region [15]. Although it would have been interesting to compare our results with estimates obtained from other Italian regions, to our knowledge, those estimates are not available.

Our study emphasizes that the observed impact of HAIs depended on indicators used and that DALYs provide a broader view of their burden. Indeed, as suggested by Cassini et al. [9], we noted a swapping between HA UTI and HA BSI if considering their incidence or DALYs. HA UTI had the lowest burden despite their high incidence, whereas HA BSI exhibited a high burden even with a relatively low incidence. For this reason, it is necessary to consider both the incidence of HAIs and their impact measured in DALYs when evaluating the needs and therefore the priorities at international, national, and regional levels. With this in mind, it is also worth stressing that about half of the four selected HAIs might be preventable through improvements in clinical practice, medical procedures, and the development and implementation of evidence-based IPC strategies [16]. Moreover, several innovative tools have been developed to reduce the incidence of HAIs and to predict patients at higher risk of adverse outcomes [17,18,19,20]. Indeed, the implementation of multimodal IPC strategies targeted to specific HAIs and groups of patients could halve the total burden of HAIs in acute care hospitals, focusing efforts to respond to the most urgent needs [16]. In 2017, Italy adopted the first national action plan on antimicrobial resistance (Piano Nazionale per il Contrasto dell’Antimicrobico-Resistenza, PNCAR 2017–2020) [21] and accordingly, the Sicilian Region implemented a regional action plan for the prevention of HAIs, AMR, and inappropriate use of antimicrobials to face the alarming AMR threat using the One Health approach and to promote effective strategies in different areas, including a regionwide multicomponent campaign using social media [22,23].

Some limitations should be considered when interpreting our results. Firstly, our analysis was limited to four HAI types and to patients aged 45 years and over, therefore generating underestimates of the total burden of HAIs in Sicily. In particular, the four HAI types were selected because of their incidence, severity, and perceived burden, the age restrictions imposed were due to the number of patients surveyed and to data availability. This highlights the need for specific studies aiming to estimate the burden of HAIs in infants, children, and young adults. Secondly, although the Rhame and Sudderth formula has been specifically designed to estimate the incidence of HAIs from prevalence data, its application was not altogether free of inaccuracies [24]. Thirdly, to our knowledge at the time of the present study, there were not studies in Sicily or Italy providing a better estimate of a correction factor for underreporting. Fourth, although multidrug resistant bacteria were associated with increased morbidity and mortality [10,25,26,27], our analysis did not estimate DALYs associated with specific antibiotic-resistant bacterium. For this reason, further research is needed to assess the burden of HAIs associated with antibiotic-resistant bacteria at national and regional level.

## 4. Materials and Methods

### 4.1. Data Source

For the current study, we used data from three consecutive regional PPSs of HAIs and antimicrobial use in acute care hospitals from Sicily: the first Sicilian PPS was launched in 2016 and was followed by two further surveys in 2017 and 2018 [7]. Each survey was in accordance with the European PPS, adopting procedures and definitions from the 5.1 version of the ECDC protocol [14]. All acute care hospitals were invited by the Sicilian Health Authority to participate in each survey and all wards were eligible for inclusion, except for accident and emergency units. According to the ECDC protocol [14], each ward carried out a single-day surveillance of patients admitted at 8 a.m. and not yet discharged at the time of the survey. There were 65 participating hospitals in 2016, 70 in 2017, and 69 in 2018. All data were confidentially collected and anonymously managed at patient level, including those related to demographic, clinical, and infectious disease information. When a patient showed evidence of being affected by an HAI, further data were collected about the type of HAI and the date of onset. All the analyses were based on anonymous information collected during the regional PPSs of HAIs in acute care hospitals implemented by the Sicilian Health Authority in the framework of a regional action plan for the prevention of HAIs, AMR, and inappropriate use of antimicrobials and hence the study did not require informed consent from participants [7].

### 4.2. Data Processing

In the current analysis, we excluded patients who had not yet completed their 45th birthday on the day of the PPS and those with missing information about age, gender and McCabe score, which was specifically used to stratify patients according to the severity of their medical conditions [28]. The McCabe score gave an indication of life expectancy and allowed stratification of patients based on whether the underlying disease was nonfatal (McCabe score 1), ultimately fatal (McCabe score 2), or rapidly fatal (McCabe score 3) [28]. Thus, from a total 18,852 participants, we excluded 1362 patients with missing data for age, gender and/or McCabe score (7.2% of the total population). Moreover, we excluded 1848 patients younger than 45 years (9.8% of the total population), because the low number of HAIs in this subsample (65 HAIs, which resulted in a prevalence of 3.5% among patients <45 years) would have produced inaccurate estimates in some age- and sex-specific strata.

The methodology of the current analysis was adapted from the BCoDE project and from Cassini et al. [8,9]. According to the definitions of the ECDC protocol [14], we first assessed the prevalence of HAP, HA BSI, SSI, and HA UTI stratified by age, gender and McCabe category for each hospital and each PPS edition. Next, we converted these measures into incidences per 100 admissions, using the Rhame and Sudderth formula [24,29]:

*I* = *P* × *LA/*(*LN* − *INT*)

where:

- *I* is the HAI incidence, that is the proportion of HAI occurrence every 100 admissions;
- *P* is the prevalence of patients with HAIs on the day of the PPS;
- *LA* is the average length of stay of hospitalized patients, extracted from each of the Sicilian PPSs;
- *LN* is the length of stay of patients with an HAI;
- *INT* is the length of stay before the onset of the HAI;
- The difference *LN* − *INT* is derived from the median number of days from HAI onset until the PPS day.

We next projected the HAI incidences from the PPSs samples to the Sicilian hospitalized population aged 45 years and above, using available data on the total number of acute care hospital discharges [30]. We applied the age, gender and McCabe distribution obtained through the analysis of each PPS edition to the regional yearly number of acute-care hospital discharges from 2016 to 2018.

To estimate the burden of HAIs, we used the BCoDE toolkit (20.0 version) by applying the same outcome trees by Cassini et al. [9] and specific model parameters and uncertainties. Outcome trees were created following a thorough literature review to represent the flow of health outcomes in the progression pathway of the disease over time, from disease development to recovery or death. Health outcomes were related to each other by transitional probabilities, their durations and disability weights [9], allowing the estimation of absolute numbers and corresponding rates (per 100,000 inhabitants equal or over 45 years) for HAI cases, attributable deaths, DALYs, YLLs, and YLDs.

For each HAI, we fitted three distinct models to account for different life expectancy across McCabe score categories (0.5 years, 3 years, or regular life expectancy obtained from Italian National Institute of Statistics). For each model, we input the gender- and age-specific yearly number of HAIs. Inputs included uncertainty intervals obtained from the variance observed across PPS editions, which were incorporated as uniform or PERT (project evaluation and review techniques) distributions [31]. As previously suggested [9,11], we applied a 1.25 under-reporting factor, and each model was run at 10,000 iterations of the Monte Carlo simulations with and without a 3.5% annual time discount rate [8]. For each outcome, estimates were reported as a median and 95% uncertainty interval (UI) of simulation outputs.

## 5. Conclusions

In conclusion, our study estimates the burden of HAIs in Sicily among patients aged 45 years and older in a total of 344 DALYs per 100,000 inhabitants. Much of this burden was attributable to HAP and HA BSI, which accounted for more than 80% of the total DALYs. Differences between our estimates and those of European and Italian PPSs encourage the estimation of the burden of HAIs at the regional level.

## Data Availability

The data presented in this study are available on request from the corresponding author.

## Supplementary Materials

The following are available online at https://www.mdpi.com/article/10.3390/antibiotics10111360/s1, Supplementary File S1. Main findings stratified by type of HAIs and McCabe score.
